# Comparative Study of the Gut Microbiota Community between the Farmed and Wild *Mastacembelus armatus* (Zig-Zag Eel)

**DOI:** 10.3390/metabo12121193

**Published:** 2022-11-29

**Authors:** Xiongjun Liu, Yuqin Fan, Tao Mo, Qingxiu Chen, Weiting Chen

**Affiliations:** Guangdong Provincial Key Laboratory of Conservation and Precision Utilization of Characteristic Agricultural Resources in Mountainous Area, School of Life Sciences, Jiaying University, Meizhou 514015, China

**Keywords:** intestinal flora, diversity, 16S rRNA sequencing, *Mastacembelus armatus*

## Abstract

Cultivated and wild fish of the same species may exhibit different characteristics, such as in their flavor, growth and development. In some wild fish species, reproductive functions may even be retarded when wild individuals are moved into cultivated conditions. The gut microbiota may be one of the reasons for these phenomena as they have been reported to play an important role in host growth and development, as well as in normal reproductive functioning. Here, we used *Mastacembelus armatus* (zig-zag eel), a freshwater fish which shows anormal reproductive function in cultivated conditions, as a model to comparatively study the diversity, structure and function of gut microbiota in cultivated and wild groups by analyzing the 16S rRNA sequence of each group’s microbiota. The results showed that Proteobacteria and Firmicutes were the dominant phyla in the gut microbiota of wild (accounting for 45.8% and 20.3% of the total number of Proteobacteria and Firmicutes, respectively) and farmed (accounting for 21.4% and 75.6% of the total number of Proteobacteria and Firmicutes, respectively) zig-zag eel. Wild zig-zag eels (Shannon = 3.56; Chao = 583.08; Ace = 579.18) had significantly higher alpha diversity than those in cultivated populations (Shannon = 2.09; Chao = 85.45; Ace = 86.14). A significant difference in the community structure of the gut microbiota was found between wild and cultivated populations. The wild zig-zag eel showed a high abundance of functional pathways in metabolism, genetic information processing and organismal system function. These results suggested that the diversity and function of gut microbiota in zig-zag eel were correlated with their diet and habitat conditions, which indicated that the management of cultivated populations should mimic the wild diet and habitat to improve the productivity and quality of farmed zig-zag eel.

## 1. Introduction

The sustainability of fisheries is an important issue in the aquaculture industry [1,2]. Fish is one of the main food sources for many people and it is rich in protein, essential fatty acids, vitamins and minerals [2,3]. Increasing aquaculture production is important in response to the declining productivity of capture fisheries and is necessary to meet the growing demand for better nutrition and economic performance [2,4]. Therefore, knowledge of the difference in the growth and development between cultivated and wild fish plays an important role in the sustainability of fisheries.

The gut microbiota co-exist and co-evolve with their host [5,6] and play an important role in host growth and development [7,8,9,10], such as in nutrition, digestion and absorption, immune system functioning, and overall health [11,12,13,14,15]. At the same time, many factors, such as host diet, genetics, physiological status and the environment, affect the gut microbiota [16,17,18,19,20]. Previous studies have investigated the gut microbiota in invertebrates, amphibians, reptiles, mammals and birds [21,22,23,24,25,26,27]. The gut microbiota of fish have received increased attention recently because they have a beneficial effect on fish growth and development [28]. They have been studied widely using traditional culture-dependent or microscopy methods [29,30,31], but many microorganisms found in fish guts have not been identified [32]. The advent of high-throughput sequencing technology (16S rRNA gene sequences) has made it possible to identify the individual microorganisms in the microbiota of many animals [33,34]; however, information on the gut microbiota of fish still lags behind that of other vertebrates [35]. In recent years, research on the gut microbiota of cultivated fish has also attracted extensive attention [35,36,37,38,39,40,41]. The gut microbiota of cultivated fish may differ from those of wild fish due to diet, physiological status and environmental factors [42,43,44,45]. Therefore, knowledge of the effects of cultivation on the gut microbiota of wild fish in conservation and rescue breeding is necessary.

The zig-zag eel is distributed mainly in Southeast Asia, including South China, inhabiting the gravelly bottoms of rivers and streams and feeding on small invertebrates and some aquatic plants [46]. It is an important economic fish in South China. It has been cultivated for several years and is well liked by people [47]. So far, however, the productivity of farming zig-zag eel is still limited. At the same time, due to climate change and the increase in multiple anthropogenic disturbances, such as dam construction, water pollution, introduction of non-native species and overfishing, the natural habitat of zig-zag eel has been destroyed and the wild population has experienced a rapid decline [48]. Captive and wild zig-zag eels may differ in diet composition due to the heterogeneity of the cultivated and natural habitats. The effect of different habitats on the gut microbiota of zig-zag eel is unclear. Here, we aimed to compare the community diversity, structure and function of the gut microbiota of captive and wild zig-zag eels using 16S rRNA gene sequencing. We hypothesized the following: (1) wild zig-zag eels have a higher gut microbiota diversity than cultivated populations and (2) the gut microbiota’s community diversity, structure and function have significant differences between cultivated and wild zig-zag eel populations. This study will provide an important reference for the aquaculture of zig-zag eel and perhaps may help to improve the aquaculture strategies.

## 2. Materials and Methods

### 2.1. Sample Collection and DNA Extraction

A total of 9 zig-zag eels, including 6 wild and 3 cultivated specimens, were used in this study, and a total of 27 gut samples were separated from these 9 specimens. Considering the complex of the wild environment, the wild sample size was then increased to make sure solid and verifiable data was obtained. We captured the wild individuals from the Bei River and collected the cultivated individuals from Jiaying University. Wild individuals mainly feed on small invertebrates and some aquatic plants from their natural environment (water temperature in the natural habitat is 17 °C) [46]. Cultivated individuals are fed on insects and kept in water temperature conditions of about 20 °C. The average body length of wild and cultivated individuals ranged from 260.0 to 320.0 mm and 240.0 to 410.0 mm, respectively. All individuals were healthy during the sampling period. We finished the sample collection from wild and farmed fish in two hours.

Due to no endangered or protected species being involved in the study, specific permission was not required for the sampling process. All necessary permits for the handling and euthanasia of animals were approved by the Animal Care and Use Committee of Jiaying University (Approval code: JYU-ACUC-2021(10)) and the Regulations for the Administration of Affairs Concerning Experimental Animals of Meizhou City. Three types of gut samples (foregut, midgut and reargut) were dissected from each individual (total gut samples were 27), and their lengths were measured using a calibrated scale and AxioVision software, as described previously with some modification [49]. Briefly, the fish were first anesthetized with MS-222 and then the full-length gut was removed. The fore, middle and rear part of the full-length gut was cut out. The contents of each part of the gut were collected in sterile centrifuge tubes and then stored at −20 °C until use. The microbial genomic DNA from each sample was extracted by using an E.Z.N.A™ Mag-Bind Soil DNA Kit (Omega, M5635-02, New York, NY, USA) following the manufacturer’s instructions. We measured the concentration of the DNA using a Qubit 4.0 (Thermo, Waltham, MA, USA) to ensure that adequate amounts of high-quality genomic DNA had been extracted.

### 2.2. 16 S rRNA Gene Sequencing

PCR amplification of the 16S rRNA genes’ V3–V4 region was performed using the forward primer (5′-CCTACGGGNGGCWGCAG-3′) and the reverse primer (5′-GACTACHVGGGTATCTAATCC-3′) [10]. The reaction was set up as follows: microbial DNA (10 ng/μL) 2 μL, amplicon PCR forward primer (10 μM) 1 μL, amplicon PCR reverse primer (10 μM) 1 μL, 2 × Hieff^®^ Robust PCR Master Mix (Yeasen, 10105ES03, China) (total 30 μL). The plate was sealed and PCR performed in a thermal instrument (Applied Biosystems 9700, Waltham, MA, USA) using the following program: 1 cycle of denaturing at 95 °C for 3 min, first 5 cycles of denaturing at 95 °C for 30 s, annealing at 45 °C for 30 s, elongation at 72 °C for 30 s, then 20 cycles of denaturing at 95 °C for 30 s, annealing at 55 °C for 30 s, elongation at 72 °C for 30 s and a final extension at 72 °C for 5 min. The PCR products were checked using electrophoresis in 2% (*w*/*v*) agarose gels in TBE buffer (Tris, boric acid, EDTA) stained with ethidium bromide (EB) and visualized under UV light.

We used Hieff NGS™ DNA Selection Beads (Yeasen, 10105ES03, Shanghai, China) to purify the free primers and primer dimer species in the amplicon product. Samples were delivered to Sangon BioTech (shanghai) Shanghai, China, for library construction using universal Illumina adaptor and index. Before sequencing, the DNA concentration and quality of each PCR product was determined using a Qubit^®^ 4.0 Green double-stranded DNA assay and bioanalyzer (Agilent 2100, Santa Clara, CA, USA). Depending on coverage needs, all libraries can be pooled for one run. The amplicons from each reaction mixture were pooled into equimolar ratios based on their concentration. Sequencing was performed using the Illumina MiSeq system (Illumina MiSeq, San Diego, CA, USA), according to the manufacturer’s instructions.

After sequencing, the two short Illumina readings were assembled by PEAR v 0.9.8 [50] according to the overlap between them. The effective tags were clustered into operational taxonomic units (OTUs) of ≥97% similarity using USEARCH v 11.0.667 [51,52]. Chimeric sequences and singleton OTUs (with only one read) were removed, after which the remaining sequences were sorted into each sample based on the OTUs. The tag sequence with the highest abundance was selected as a representative sequence within each cluster. Bacterial and fungal OTU representative sequences were classified taxonomically by blasting against the RDP Database (http://rdp.cme.msu.edu/misc/resources.jsp; accessed on 1 June 2016) and UNITE fungal ITS Database (http://unite.ut.ee/index.php; accessed on 29 October 2018), respectively.

### 2.3. Data Analysis

The alpha diversity indices (including Chao1, Simpson and Shannon indices) were quantified in terms of OTU richness. To assess sample adequacy, rarefaction curves of the observed numbers of OTUs were constructed. All alpha diversity indices were calculated with Mothur v 3.8.31 [53]. The OTU rarefaction curve and rank abundance curves were plotted in R 3.5.0 [54]. To estimate the diversity of the microbial community of the sample, we calculated the within-sample (alpha) diversity using a T test for two groups, and multiple group comparisons were made using an ANOVA test. The Venn diagram was constructed using the R 3.5.0 [54] for drawing. Beta-diversity evaluates differences in the gut microbiota among samples and is normally combined with heatmap analysis and non-metric multidimensional scaling (NMDS) to obtain visual representations. These analyses were visualized using the R vegan package [55], and finally, the inter-sample distances were presented as scatterplots. Difference comparison was used to identify features with significantly different abundances between groups using LefSe v 1.1.0 [56]. Correlation coefficients and *p*-values between communities/OTUs were calculated using SparCC v 1.1.0 [57], and correlation matrix heatmaps were drawn using the R corrplot package. Functional prediction analysis of gut microbiota was performed using PICRUSt v1.1.4 [58] by comparing existing 16S rRNA gene sequencing data with a microbial reference genome database of known metabolic functions, enabling the prediction of gut microbiota metabolic functions.

## 3. Results

### 3.1. Composition of the Gut Microbiota in the Cultivated and Wild Zig-Zag Eels

**Figure 1:**
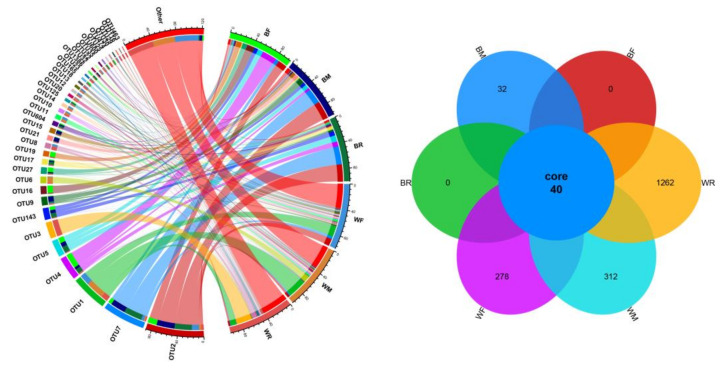
Hierarchical cluster analysis and Venn diagram at the OTU level of gut microbiota in cultivated and wild *Mastacembelus armatus*. The hierarchical cluster analysis and Venn diagram were constructed using R 3.5.0 [59] for drawing.

A total of 3449 OTUs were obtained at 97% sequence similarity (Figure S1), belonging to 31 phyla, 73 classes, 128 orders, 264 families and 566 genera. A total of 40 OTUs were shared by cultivated and wild zig-zag eels, whereas 32 OTUs and 1852 OTUs were unique to the cultivated and wild groups, respectively (Figure 1). A total of 4 phyla, 21 families and 29 genera were shared by the cultivated and wild groups; 0 phyla, 2 families and 2 genera were specific to the cultivated group and 3 phyla, 45 families and 141 genera were specific to the wild group (Table S1; Figure S2).

**Figure 2:**
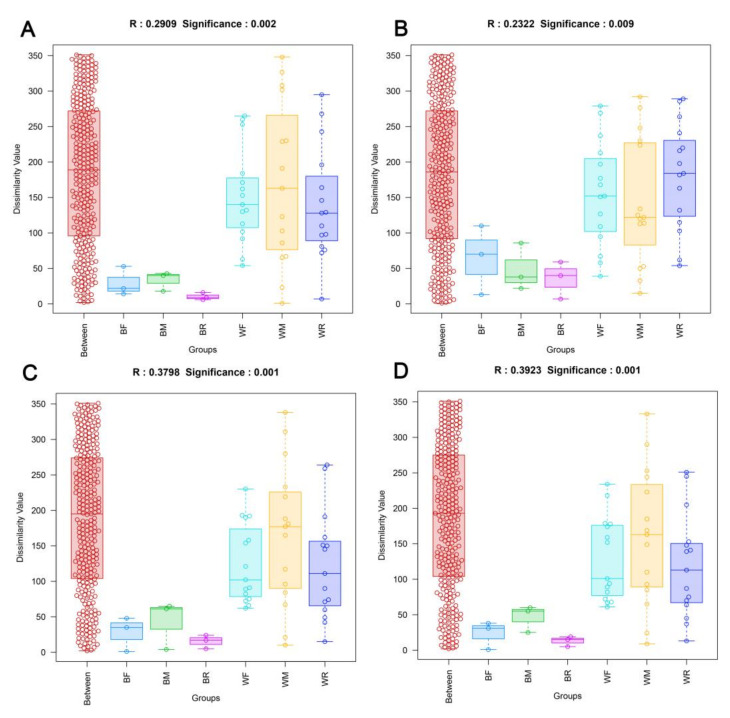
ANOSIM analysis at the OTU (**A**), phylum (**B**), family (**C**) and genus (**D**) levels of gut microbiota composition in cultivated and wild *Mastacembelus armatus*.

**Figure 3:**
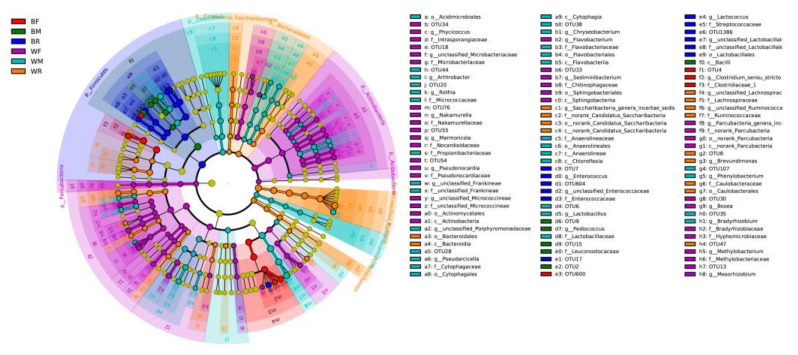
LEfSe (LDA Effect Size) analysis of the gut microbiota in cultivated and wild *Mastacembelus armatus*.

The compositions of the gut microbiota in the cultivated and wild groups were significantly different at the OTU, phylum, family and genus levels (ANOSIM; *p* < 0.05; Figure 2). Firmicutes in the cultivated group and Proteobacteria in the wild group were the dominant phyla, followed by Proteobacteria in the cultivated group and Firmicutes and Bacteroidetes in the wild group (Figure S2). At the family level, the main gut microbiota included Streptococcaceae, Enterobacteriaceae and Enterococcaceae in the cultivated group, and Ruminococcaceae and norank Gammaproteobacteria incertae sedis were dominant in the wild group (Figure S2). At the genus level, the main gut microbiota included *Lactococcus* in the cultivated group and *Candidatus Carsonella* in the wild group (Figure S2). The LEfSe analysis showed that Firmicutes and Proteobacteria were the most important taxa contributing to the differences in the gut microbiota between the cultivated and wild groups (Figure 3).

### 3.2. Diversity of the Gut Microbiota in the Cultivated and Wild Zig-Zag Eels

A significant difference was found in the alpha-diversity of the gut microbiota (*p* < 0.05; Table 1). Wild zig-zag eels had higher alpha-diversity in their gut microbiota than those in the cultivated group (Table 1).

**Figure 4:**
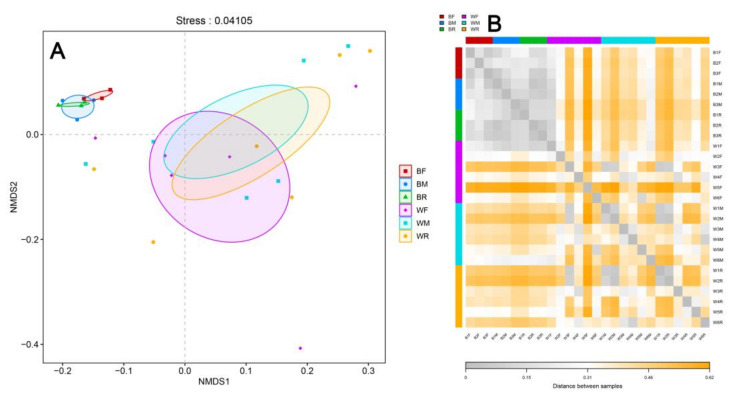
Heatmap analysis (**B**) and non-metric multidimensional scaling (NMDS) analysis (**A**) of the gut microbiota in cultivated and wild *Mastacembelus armatus*.

Heatmap analysis and non-metric multidimensional scaling (NMDS) showed that the community structures of the gut microbiota of zig-zag eels at the OTU, phylum, family and genus levels were both divided into two groups, with the first cluster being formed by the wild group and the second cluster being formed by the cultivated group (Figure 4 and Figure S3).

### 3.3. Gut Microbiota Functional Profile Prediction

**Figure 5:**
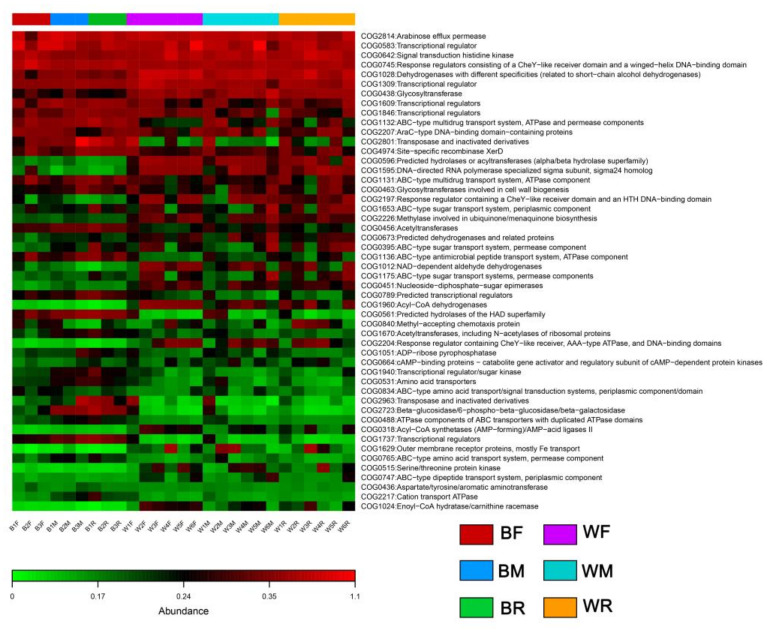
Differences in the functional profiles in pathways of the gut microbiota in cultivated and wild *Mastacembelus armatus*.

A total of 50 functional categories in cultivated and wild groups were predicted in this study (Figure 5). There were 26 functional categories in cultivated and wild groups with significant differences (ANOVA, *p* < 0.05). In cultivated groups, the pathways of arabinose efflux permease, transcriptional regulators, signal transduction histidine kinase and response regulators were more enriched than other pathways (Figure 5). In wild groups, the pathways of arabinose efflux permease, transcriptional regulators, signal transduction histidine kinase, response regulators, dehydrogenases with different specificities and glycosyltransferase were more enriched than the other pathways (Figure 5).

## 4. Discussion

Some studies showed that Firmicutes and Proteobacteria were the dominant phyla in the gut microbiota of fish [60], reptiles [61], mammals [62] and birds [63]. Firmicutes and Proteobacteria have effects on the metabolism and immune function of host [61,64,65,66]. For example, Firmicutes can help digest and absorb proteins [67,68,69]. The Proteobacteria observed in the fish gut samples have been associated with fish gastroenteritis [64,65]. In this study, Firmicutes and Proteobacteria were also the most important taxa contributing to gut microbiota in both cultivated and wild groups, indicating these taxa play an important role in maintaining the relative stability of the gut microbiota of zig-zag eels. In addition, the increased presence of Firmicutes and Proteobacteria in the cultivated and wild groups indicates that the gut microbiota of this species are more efficient at digesting food to help hosts obtain energy, which is a favorable adaptive strategy for living in complex habitats [63,70]. At the genus level, the observed prominence of *Lactococcus* in the gut microbiota of the cultivated group and of *Candidatus Carsonella* in that of the wild group is inconsistent with reports by others including *Aeromonas* spp. [71,72], *Cetobacterium* spp. [73] and *Cetobacterium somerae* [74].

A significant difference was found in the community structure of the gut microbiota between the cultivated and wild zig-zag eels. Wild zig-zag eels had higher alpha-diversity and a higher number of OTUs in their gut microbiota than those of the cultivated group. Previous studies have revealed that the community structure of the gut microbiota of freshwater fish differs significantly [42,43,44] depending on their habitat and diet [75,76]. The community structure of the gut microbiota is affected by the complex direct and indirect interactions of many external (e.g., habitat difference) and internal (e.g., age, diet) factors [9,32,35,77]. The habitat characteristic may affect the composition of the gut microbiota of aquatic organisms [66,78]. For example, there are significant differences in the composition of the gut microbiota between marine and freshwater fish [35]. Environmental differences in the gut microbiota of fish have been previously shown in wild and cultivated fish species [79,80]. The wild zig-zag eel investigated in this study inhabits rivers and streams with higher habitat heterogeneity [48], which may allow for a higher gut microbiota diversity and a greater number of different taxa in the wild group. Therefore, the diversity and structure of the gut microbial community differed significantly as a result of inhabiting different habitats, indicating that the wild habitat has an obvious effect on zig-zag eels’ gut microbiota. In addition, diet composition differences also play an important role in gut microbiota diversity [37,38,39,40]. Cultivated zig-zag eels in this study feed mainly on insects and are fed adequate food at regular intervals, whereas the wild group mainly eats small invertebrates and some aquatic plants and individuals may have different diets [46]. Therefore, combining our analysis with biological (e.g., diet) and environmental factors (e.g., habitat difference, human pressure) would help further define the community structure of the gut microbiota of zig-zag eels.

Functional prediction can link the structure and function of gut microbiota and better elucidate its pathogenesis [81,82]. For example, a previous study showed that the core gut microbiota on the skin of *Plethodon cinereus* was closely related to immunomodulation [82]. Metabolic processes, such as antibacterial immunity, lysosomes and peroxidase, were weakened in diseased animals [82]. Diet differences affect the function of the gut microbiota in the host [83]. In this study, we found that the gut microbiota community in cultivated and wild groups of zig-zag eels exhibited various levels of functional diversity. The abundance of Proteobacteria in the gut microbiota of the wild group was significantly higher than that in cultivated groups. Proteobacteria have a number of specific persistent genes that are devoted to RNA processing and degradation, as well as outer membrane and lipopolysaccharide synthesis [84]. This might jointly lead to the fact that the predicted pathways of arabinose efflux permease, transcriptional regulators, signal transduction histidine kinase, response regulators, dehydrogenases with different specificities and glycosyltransferase are more enriched in wild groups. The living conditions of the cultivated group may affect the relative abundance of potential pathogens in the gut microbiota [85]. It is worth observing that the pathways related to arabinose efflux permease, transcriptional regulators, signal transduction histidine kinase and response regulators were more enriched in cultivated groups. This might be related to the dominant Firmicutes because it has an influence on the metabolism and immune function of the host [60,86].

## 5. Conclusions

In conclusion, there were significant differences between cultivated and wild zig-zag eels in terms of the community structures and diversity of their gut microbiota. Wild zig-zag eels had a higher diversity of gut microbiota and had more metabolism, genetic information processing and organismal systems pathways when compared with the cultivated group. These differences may be correlated with the habitat and diet differences between cultivated and wild populations. This study can provide a reference for researchers to further understand the correlation between the gut microbiota of zig-zag eel and its habitat. Finally, according to our results, we would recommend that fishermen try to mimic the natural conditions such as food, light and water when farming zig-zag eels to improve the product quality and productivity of the aquaculturing operation.

## Figures and Tables

**Table 1:** Sequences, OTUs and alpha-diversity indices of gut microbiota of cultivated and wild *Mastacembelus armatus*. BF: foregut of cultivated individual; BM: midgut of cultivated individual; BR: reargut of cultivated individual; WF: foregut of wild individual; WM: midgut of wild individual; WR: reargut of wild individual.

	BF	BM	BR	WF	WM	WR	*p* Value
Sequences	43,598 ± 8902	62,634 ± 7627	45,974 ± 12842	46,397 ± 23293	55,732 ± 10324	49,985 ± 12,155	0.54
OTUs	51± 6	89 ± 5	70 ± 20	498 ± 251	441 ± 354	728 ± 468	0.02
Shannon	2.30 ± 0.52	2.03 ± 0.47	1.95 ± 0.27	3.74 ± 0.72	3.08 ± 1.36	3.86 ± 1.74	0.08
Chao	56.64 ± 9.69	98.58 ± 11.07	101.12 ± 32.71	536.03 ± 258.75	465.61 ± 371.51	747.60 ± 470.24	0.02
Ace	60.93 ± 14.02	99.77 ± 13.02	97.72 ± 36.01	532.51 ± 244.49	467.70 ± 368.59	737.34 ± 469.79	0.02
Simpson	0.15 ± 0.08	0.24 ± 0.14	0.21 ± 0.06	0.12 ± 0.11	0.21 ± 0.21	0.17 ± 0.19	0.87

## Data Availability

All raw sequences were deposited in the NCBI Sequence Read Archive (https://www.ncbi.nlm.nih.gov/; accessed on 1 January 2022) under accession number SRA Accession no. PRJNA885226.

## Supplementary Materials

The following supporting information can be downloaded at: https://www.mdpi.com/article/10.3390/metabo12121193/s1, Figure S1: Rarefaction curves for OT number of gut microbiota from the cultivated and wild Mastacembelus armatus. Data were obtained using a threshold of 97%. Figure S2: Hierarchical cluster analysis and Venn diagram of gut microbiota at phylum (A,B), family (C,D) and genus (E,F) level. Figure S3: Difference in gut microbiota community structure between cultivated and wild Mastacembelus armatus using non-metric multidimensional scaling (NMDS) and heatmaps analysis at phylum (A,B), family (C,D) and genus (E,F). Table S1: The composition of the gut microbiota in the cultivated and wild zig-zag eels.
