# Supplementary material for: Comparative Study of the Gut Microbiota Community between the Farmed and Wild Mastacembelus armatus (Zig-Zag Eel)
Source: Metabolites. 2022 Nov 29;12(12):1193. doi: 10.3390/metabo12121193 (PMC9781078; doi:10.3390/metabo12121193)
Supplement: Supplementary file 1 [file metabolites-12-01193-s001.zip › Table S1.pdf]

Table S1. The composition of the gut microbiota in the cultivated and wild zig-zag eels

	phylum	Class	order	family	genus
BF	5	7	13	27	40
BM	11	28	43	82	115
BR	11	15	26	53	75
WF	28	61	108	210	378
WM	26	62	111	213	418
WR	31	66	115	230	486
