# Supplementary figures and images for: Comparative Study of the Gut Microbiota Community between the Farmed and Wild Mastacembelus armatus (Zig-Zag Eel)
Source: Metabolites. 2022 Nov 29;12(12):1193. doi: 10.3390/metabo12121193 (PMC9781078; doi:10.3390/metabo12121193)

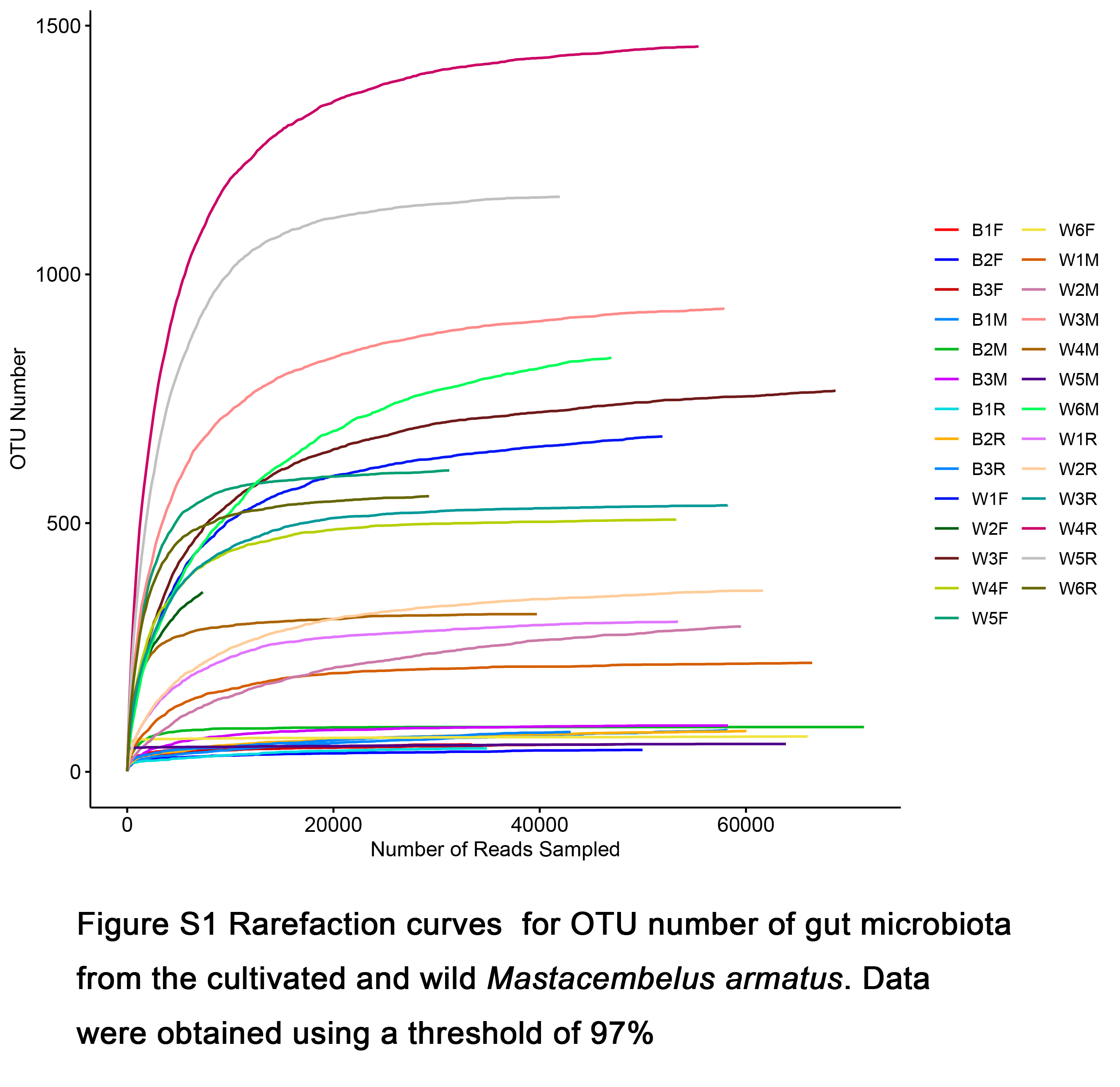

Supplement: Supplementary file 1 [file metabolites-12-01193-s001.zip › Figure S1.jpg]

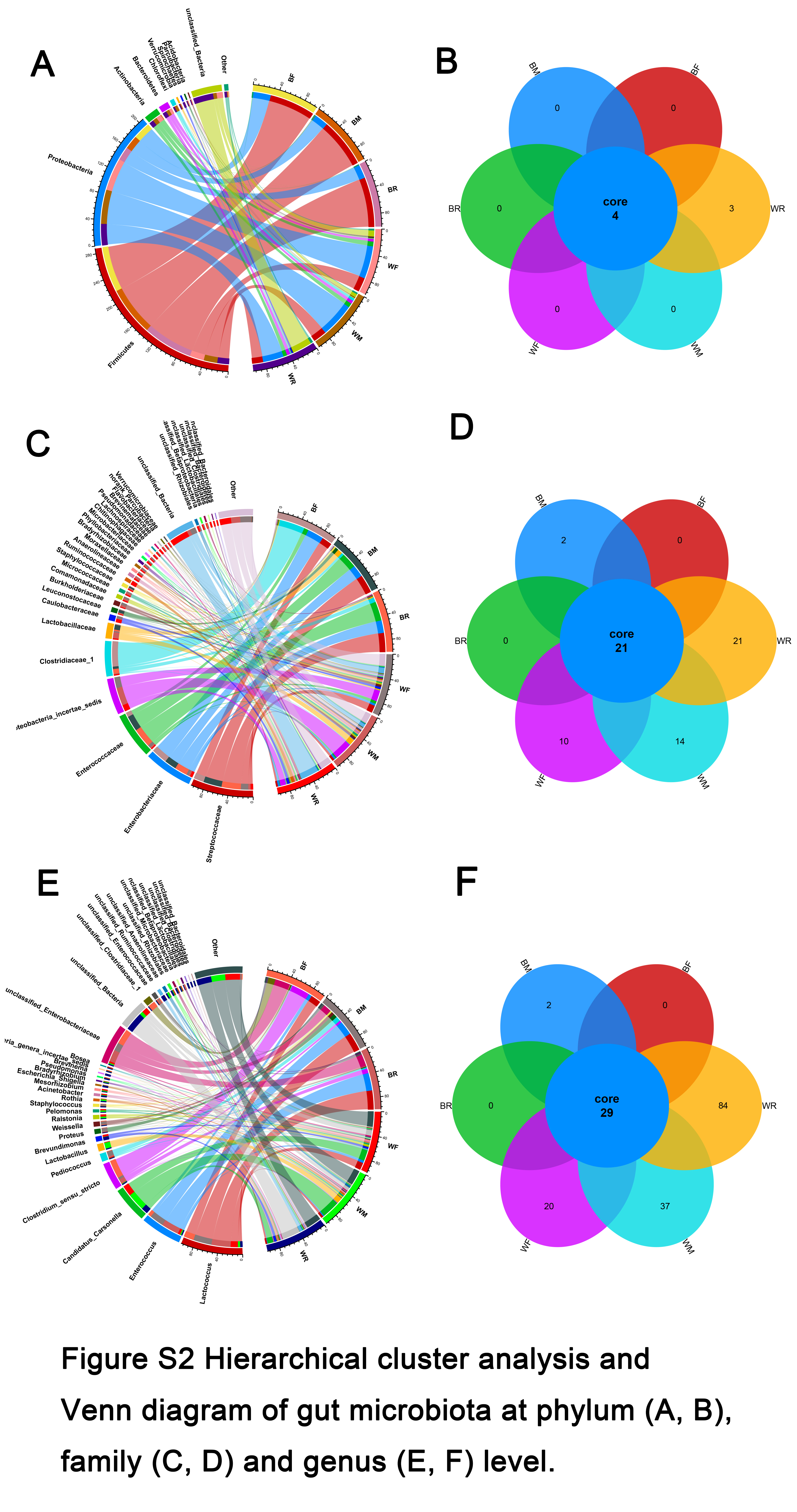

Supplement: Supplementary file 1 [file metabolites-12-01193-s001.zip › Figure S2.jpg]

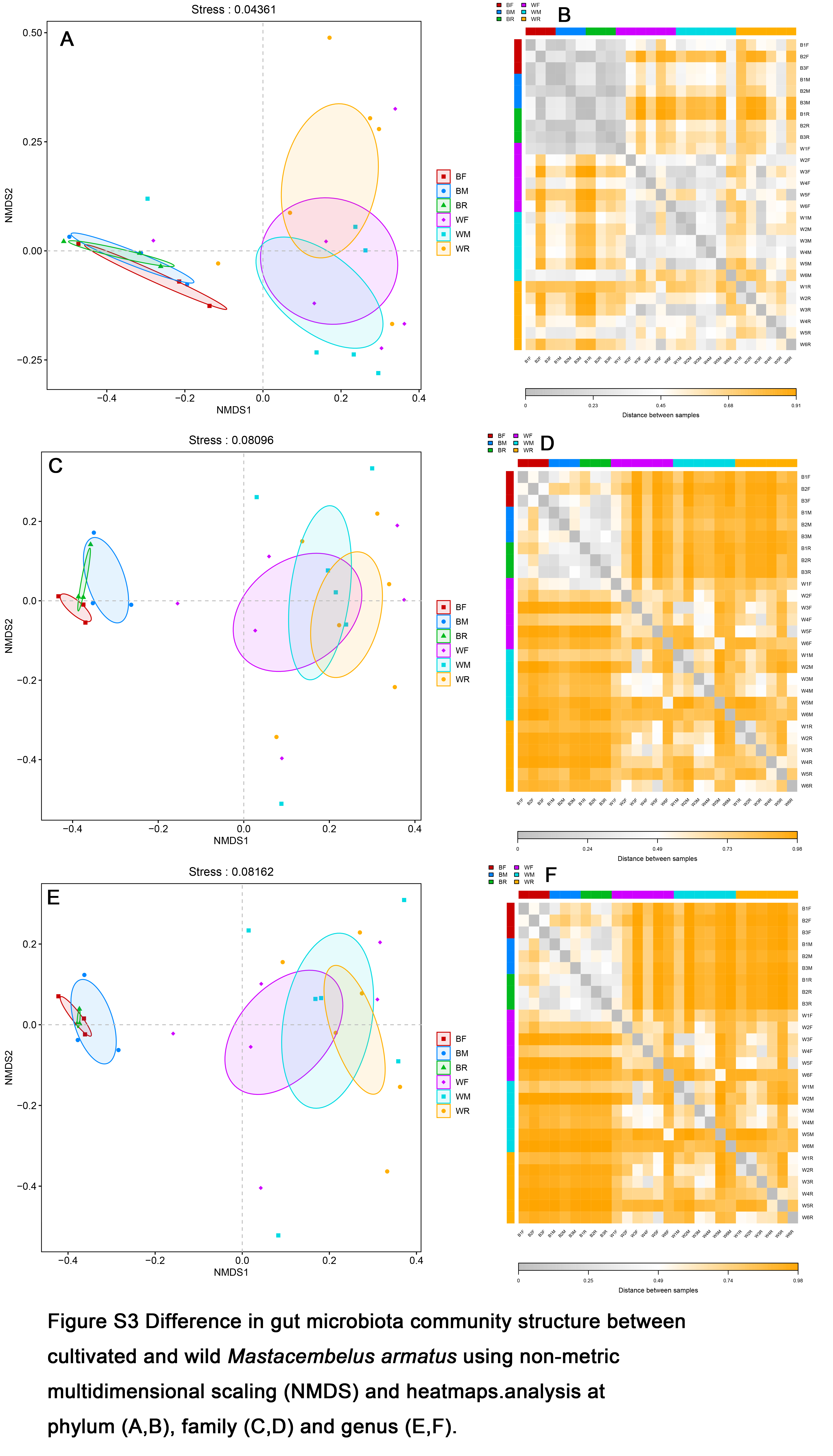

Supplement: Supplementary file 1 [file metabolites-12-01193-s001.zip › Figure S3.jpg]
